# Economy in the Poor-Law Infirmary

**Published:** 1907-05-11

**Authors:** 


### ECONOMY IN THE POOR-LAW INFIRMARY.

By the Author of "Hospital Expenditure: The Commissariat."

The store-room has only quite recently secured recognition at the hands of the hospital architect. In old Georgian and Queen Anne domestic architecture it is true that the kitchen accommodation is excellent; but the older hospitals and nearly all the Poor-law infirmaries are lamentably deficient in every convenience, and can at best show whitewashed cellars with gratings instead of properly planned domestic premises. It is hardly too much to say that every institution ought to begin its existence by making exact provision for the reception and distribution of the stores. Whatever its functions, the institution is from the beginning a consumer. Whatever its income, a large proportion of the total must be spent on goods which are hereafter to be re-distributed, and whether its affairs will be regulated with economy or extravagance depends solely on the degree of efficiency attained in buying and storing these goods.

Who would open a shop without ascertaining what accommodation is afforded for the stock in trade? Yet many Poor-law infirmaries deal with a daily bulk of provisions far exceeding that of the ordinary retail trader, and are so far from realising their responsibilities that they are content with larders hardly adequate for an ordinary middle class household, and allow their daily supplies to be delivered direct into the general kitchen.

In the well-organised institutions the premises devoted to goods perishable and imperishable are far more than store-rooms. They are offices in which an immense amount of important work has to be got through. This work falls naturally under three headings—receiving, storing, and issuing—and unless all three branches of the work be well performed, there will be a leak in expenditure, although it may not be one which is readily detected.

### The Receiving-rooms.

Whether the institution be large or small, no article should be delivered to it without passing under the scrutiny of a trained eye, and rendering account of quality, weight, and measure. The instinct of the old pensioner who may be seen to turn over her two-penny fragment of meat again and again before she can make up her mind to the purchase is a sound one. It is carried into practice by every careful housekeeper. Only in the institution where the health of hundreds depends on the quality of the provisions are they received with no precautions beyond a hurried weighing, and under conditions which make any real examination an impossibility. It is more expeditious for perishable goods to be received in larders adapted for their separate storage. These should be at least six in number; for meat; for fish and poultry; for milk and butter; for bread; for fresh vegetables; for eggs, cheese, and bacon. In large institutions the steward, in small ones the secretary or housekeeper, is responsible for checking the weight and examining into the quality of every joint, every fish, every loaf of bread, every head of greens. Ample time should be allowed for carrying out this business carefully. The results should be noted in a book kept for the purpose, which should be regularly presented for inspection by the Weekly Board. The person entrusted with this duty ought to be trained to distinguish between good and bad provisions, and should be expected to show some evidence of ability in this connection. Ninety per cent. of bad institution housekeeping results from the fact that no sort of training is ever given or required in the business of choosing provisions. A clever steward or housekeeper picks it up by practice, but few indeed are those fitted to go behind a dishonest contractor and expose the ingenious ways through which he manages, in spite of underselling all competitors, to secure a gigantic profit. The very fact, however, of keeping a regular record of the condition of all provisions received helps to lay the foundation of sound knowledge. However desirable it may be for the cook to assist in this daily inspection, it cannot be too strongly insisted upon that it should be done under the keen and practised eye of someone in authority, responsible for keeping an exact daily record of provisions as regards weight, condition when received, and quality. A separate receiving-room must be provided for dry goods of every description, nor should any article be delivered to any functionary in the hospital until it has been officially checked and entered as correct in all particulars in the books kept for that purpose. All groceries, all household necessaries, all linen or materials for uniform, all drugs, dressings, and surgical appliances should undergo the same careful process of examination, of weighing, or measuring. In the official Magasin Central from which Parisian hospitals receive all their supplies, every article is subjected to scrutiny by experts, and this extends in the case of bales of goods to the separate stretching and testing of every yard of material. The whole business is performed with incredible thoroughness and expedition, and each contractor understands the necessity for fulfilling his obligations to the very letter. Such a system, if it could be enforced in London Poor-law infirmaries, would go far towards justifying Mr. Asquith's surmise that a good start with old-age pensions could be built up out of the savings it is possible to effect in the administration of Poor-law relief. But with regard to the two essentials in this connection, well-planned premises and a well-paid steward, the Guardians of the Poor are in most instances inflexible. They decline to sanction additional expenditure in either direction. It is impossible that goods received into dark, ill-ventilated cellars can ever undergo a proper scrutiny. It is the duty of Poor-law Guardians to make themselves fully acquainted with the facts, and to insist upon proper facilities being afforded to their steward for carrying out his responsible duties. They will probably be surprised at the accommodation available, even in some institutions where the administration in other respects is excellent.

